# Non-Curative Endoscopic Submucosal Dissection: Current Concepts, Pitfalls and Future Perspectives

**DOI:** 10.3390/jcm14072488

**Published:** 2025-04-05

**Authors:** João Santos-Antunes

**Affiliations:** 1 Gastroenterology Department, Porto WGO Training Center, Centro Hospitalar S. João, 4200-319 Porto, Portugal; joao.claudio.antunes@gmail.com; 2 Department of Medicine, Faculty of Medicine, University of Porto, 4200-319 Porto, Portugal; 3 IPATIMUP—Institute of Molecular Pathology and Immunology, University of Porto, 4200-135 Porto, Portugal

**Keywords:** endoscopic submucosal dissection, non-curative, digestive tract neoplasia, early cancer, endoscopic treatment

## Abstract

Endoscopic submucosal dissection (ESD) is very effective for the treatment of digestive tract neoplasia. However, it is very demanding, with a long learning curve, and, therefore, a significant rate of non-curative resections is expected, considering lesion characteristics, location, and endoscopist experience. The management of patients after a non-curative ESD is not definitely established. It must consider patients’ comorbidities and expected survival, as well as the morbidity and mortality of complementary treatments such as surgery, chemotherapy, or radiotherapy. On the other hand, there is a window of opportunity to offer those additional treatments to complete neoplastic treatment and give patients an oncological cure. This decision is sometimes difficult, since the diverse histological criteria that define a non-curative ESD do not have the same weight regarding residual risk and oncological progression. The prediction of residual lesion would be paramount to decide whether to refer patients to surgery; nowadays, this prediction is far from perfect, since most of the patients that undergo surgery due to a non-curative ESD do not have residual neoplasia in the surgical specimen. In this review, ESD curativeness and the management of non-curative ESDs performed for esophageal, gastric, and colorectal lesions will be addressed.

## 1. Introduction

The gastrointestinal tract is the organ system bearing the highest cancer incidence and mortality. Data from Globocan 2020 [1] estimate a cumulative number of deaths in the world of nearly 2,250,000, including esophagus, stomach, and colorectum, corresponding to more than 22% of the total deaths by cancer.

To reduce the burden of gastrointestinal cancer, we need to carry out important preventive measures, avoiding known risk factors such as smoking or alcohol, and to improve *Helicobacter pylori* eradication rates. Also, it is paramount to increase the efficacy of screening strategies aiming to detect and treat premalignant lesions, avoiding its progression towards malignancy. The detection of premalignant or malignant lesions at an early stage will allow an endoscopic curative resection without compromising survival, while avoiding the morbidity and mortality of classical surgical procedures.

In this narrative review, the indications of one of the most valuable endoscopic resection techniques for the treatment of early digestive tract neoplasia, endoscopic submucosal dissection (ESD), will be explained. Furthermore, the complex subject of the management of patients after a non-curative ESD (NC-ESD) will be thoroughly discussed.

## 2. The Development of ESD and Its Establishment as a Mainstay Treatment for Gastrointestinal Early Neoplasia

**Figure 1:**
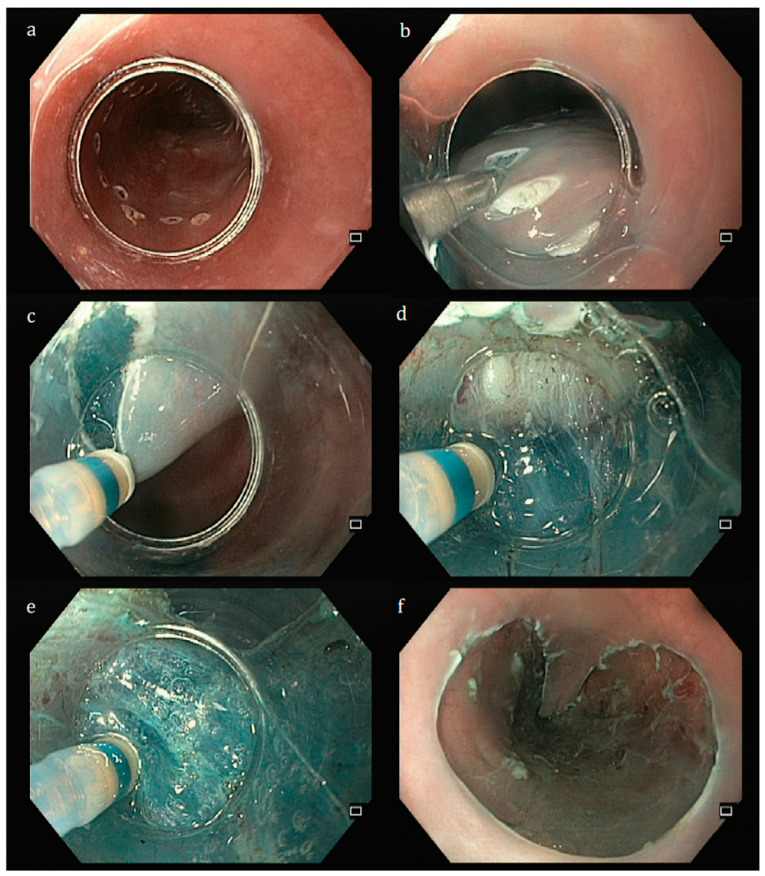
Steps for endoscopic submucosal dissection. (**a**)—circumferential marking using the tip of the dual-knife; (**b**)—submucosal injection to create a cushion between the lesion and the muscularis propria; (**c**)—mucosal incision using the dual-knife; (**d**)—submucosal dissection; (**e**)—further submucosal injection using the same device; (**f**)—final eschar. Author’s own images.

The story of this technique started in Japan more than 30 years ago. In 1988, Hirao et al. [2] developed an endoscopic resection technique using local injection of hypertonic saline and epinephrine, with submucosal dissection using a needle-knife. In 1995, Hosokawa and Yoshida added an insulated ceramic tip to the needle-knife in order to decrease the risk of perforation, developing the insulate-tip knife (IT-knife) [3], used for rectal and gastric lesions by Gotoda et al. in 1999 [4] and Ono et al. in 2001 [5]. At this time, this procedure was still called Endoscopic Mucosal Resection (EMR). In 2002, a new “aggressive EMR method” for the upper gastrointestinal tract, the hook-knife method, was developed by Oyama et al. [6,7]. In 2004, Yahagi et al. developed a new technique by performing submucosal dissection (ESD) using the tip of an electrosurgical snare [8], which was the predecessor of the flex-knife [9]. In 2011, Yahagi et al. developed and published the first experience with an improved version of the flex-knife, the dual-knife [10] (Figure 1). In Europe, ESD practice started with pilot studies in a few centers in the mid-2000s, while training programs for Western endoscopists using animal models have been developed particularly since 2011 [11].

ESD indications have expanded throughout the gastrointestinal tract, mainly esophagus, stomach, colon, and rectum, but also to other challenging locations such as duodenum and small bowel or pharynx. Furthermore, several ESD-related techniques, such as Peroral Endoscopic Myotomy (POEM), Submucosal Tunneling Endoscopic Resection (STER), Knife-assisted Resection/hybrid Endoscopic Mucosal Resection (EMR)-ESD techniques, Endoscopic Intermuscular Dissection and endoscopic–laparoscopic collaborative techniques, have been developed.

Nowadays, ESD has reached an incredibly vast experience in the East, such as in Japan, Republic of Korea, or China. In the Western setting, the implementation and development of this technique have been much slower, due to several aspects, such as policy strategies, less experienced endoscopists, and a much lower number of gastric lesions, not only by a lower incidence of gastric cancer but also due to a lack of systematic screening, opposite to what is verified in the high-incidence areas of the southern east parts of Asia. However, its widespread use is clearly developing at a higher pace in Europe in recent years.

## 3. ESD Along the Gastrointestinal Tract

### 3.1. Esophagus

**Figure 2:**
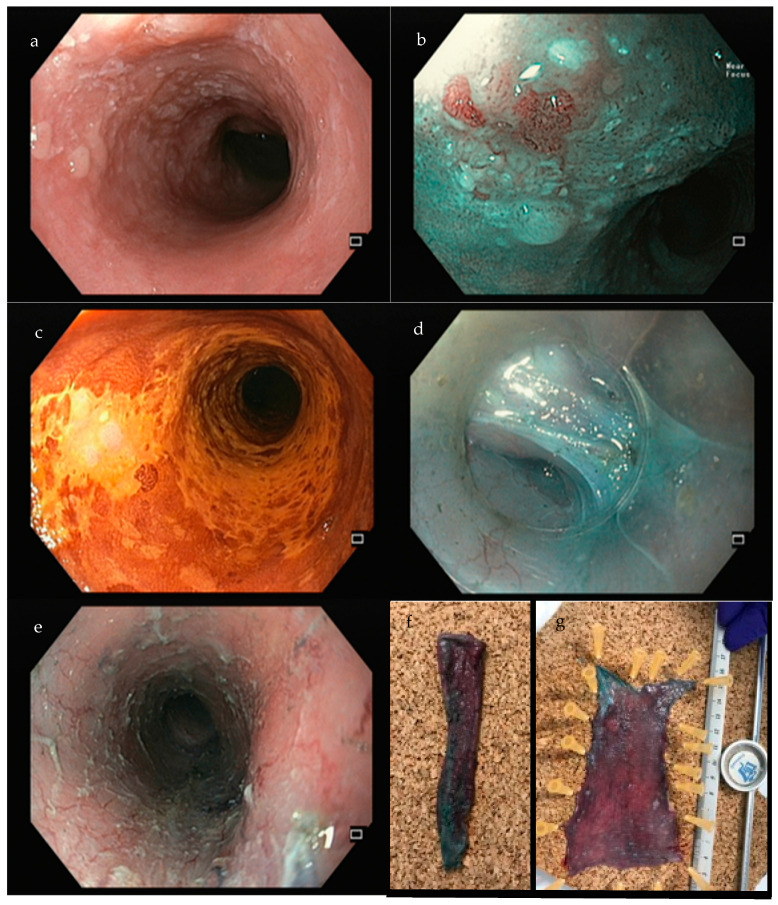
Example of an ESD performed for an esophageal SCC. (**a**)—white-light; (**b**)—narrow band imaging; (**c**)—lugol staining; (**d**)—submucosal dissection; (**e**)—final eschar, after circumferential dissection; (**f**)—cylinder of ESD specimen; (**g**)—fixation of the specimen in a cork plate. Author’s own images.

ESD is the first-line endoscopic treatment of early neoplasia in the squamous epithelium (Figure 2). Overall, ESD offers a higher complete resection rate, a higher 5-year disease-free survival rate, and a lower recurrence rate compared to EMR, being equal to surgery concerning overall survival or disease-free survival, with better safety profiles, particularly among intramucosal or sm1 submucosal squamous cell cancer (SCC) [12]. According to European Society of Gastrointestinal Endoscopy (ESGE) guidelines, it is the first-line treatment for esophageal dysplasia or SCC, particularly if limited to the mucosa or, in selected cases, with superficial invasion of the submucosa.

Japanese guidelines [13] state that endoscopic resection techniques are indicated for intramucosal m1 or m2 SCC, even if fully circumferential if smaller than 5 cm longitudinally. For m3/sm1 SCC, endoscopic resection may still be indicated, if non-circumferential, providing the real risk of requiring further treatment is explained to the patient. Patients with R0 resection of intramucosal SCC without lymphovascular invasion are considered cured, while in those with submucosal invasion or lymphovascular permeation, complementary treatment is advisable.

In the West, ESD is safe and effective for SCC, but studies have reported a curative resection only in nearly half of the cases [14], with significant lower overall survival in those with a non-curative resection [15]. In the case of circumferential lesions, ESD may also be a feasible treatment in selected patients, but with a high risk of a non-curative resection and difficult-to-treat strictures [16].

ESD for Barrett’s esophagus is also accepted, but good results have been described using EMR-derived techniques. As expected, ESD is more efficient and warrants more en bloc and curative resections, with less recurrence and need for repeated treatments, with a very good safety profile [17,18]. Generally, it is accepted that ESD may be specially indicated in particular circumstances, namely, upon the suspicion of malignant lesions with submucosal invasion, nodular lesions, or scarring [12].

### 3.2. Stomach

ESD is the mainstay of endoscopic treatment of gastric dysplasia and early cancer, since it offers a higher rate of curative resections and less recurrence, at the expense of longer procedural times and higher perforation rates [12,19,20,21] compared to EMR. It shows higher en bloc, complete, and curative resection rates and less local recurrence [21].

Absolute indications for ESD include benign or intramucosal differentiated and non-ulcerated adenocarcinomas of any size, intramucosal undifferentiated adenocarcinoma less than 2 cm, and intramucosal, differentiated, and ulcerated lesions less than 3 cm: the classical expanded criteria for ESD of the first edition of the Japanese guidelines [22] were in 2021 recategorized as absolute indications [23], and are also present in the most recent European guidelines.

Compared to surgery, endoscopic resection has a lower incidence of adverse events and length of hospital stay and a similar overall survival and cancer-specific survival, despite a higher rate of recurrence and lower complete resection rate [24]; it is also useful for the treatment of undifferentiated-type early gastric cancer, with similar overall survival despite a higher rate of recurrence and less disease-free survival [25]. It should be noted that, in the event of a non-curative ESD, this procedure does not negatively impact the short- and long-term outcomes of the gastrectomy [26].

**Figure 3:**
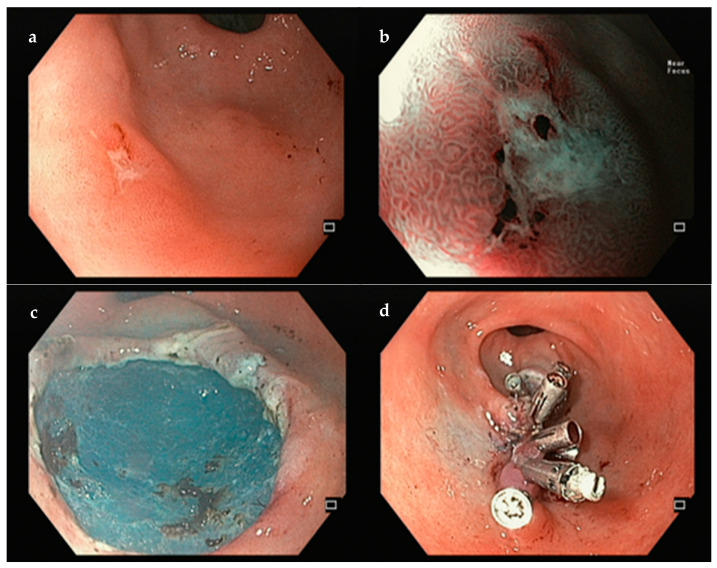
Example of an ESD performed for a gastric high-grade dysplasia. (**a**)—white-light; (**b**)—narrow band imaging; (**c**)—final eschar; (**d**)—clipping the mucosal defect for closure was decided due to patient’s high risk of late bleeding. Author’s own images.

In the West, results are now found to be similar to the East [27,28,29], and its advantages over gastrectomy have been documented [30] (Figure 3).

### 3.3. Colon and Rectum

The best treatment for colorectal lesions, especially Lateral Spreading Tumors (LSTs) above 2 cm, is still controversial. European guidelines recommend ESD in lesions suspicious of harboring malignancy, while piecemeal EMR would be sufficient for the remaining [12,31], but with the need of several endoscopic procedures due to the higher risk of recurrence. Some techniques have been described as aiming to decrease the rate of recurrence after a piecemeal EMR, such as margin marking before the resection [32] or margin ablation with argon plasma [33] or snare tip coagulation [34] after the resection. Underwater EMR has been shown to raise en bloc and R0 resection rates compared to conventional EMR, with similar procedural time and adverse events [35].

**Figure 4:**
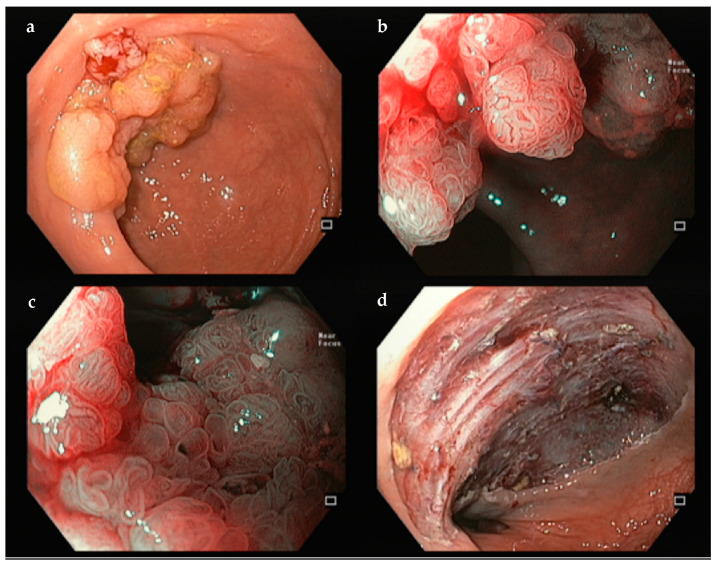
Example of an ESD performed for a rectal high-grade dysplasia. (**a**)—white-light; (**b**,**c**)—narrow band imaging; (**d**)—final eschar. Author’s own images.

Colorectal ESD is a specialized and demanding technique (Figure 4). A meta-analysis [36] showed that, overall, results from Asian studies are better than from non-Asian studies, and worse in centers with low number of ESDs and with a lack of organized training. A lower rate of R0 resection in the rectum than in the colon (75% vs. 85%) was also found, probably due to the fact that rectal lesions are frequently the first lesions to be selected in the ESD learning curve in the West, due to the relative infrequency of gastric lesions.

In Japan, all the lesions are characteristically removed in one piece, so the chosen technique will relate to the capability of en bloc resection: smaller lesions can be retrieved in a single piece with a snare, while all the others should be resected by ESD. Nevertheless, Japanese guidelines [37] stated that most colorectal neoplasms are adenomas, and therefore can be cured using EMR techniques, provided that the area with the most advanced histology is resected en bloc, with the remaining adenoma resected by piecemeal. However, it is stated that complete en bloc resection is indicated for early colorectal carcinoma regardless of the tumor size. It should be noticed that carcinoma definition is different between Japan and Western countries, and some early carcinomas in Japan are considered high-grade dysplastic lesions in the West [38].

In the West, most of the LSTs without high-risk features upon endoscopic evaluation are managed by piecemeal EMR. However, this is far from being a consensus, since some authors defend an almost universal application of ESD in the colon and rectum, to avoid histological information loss due to a piecemeal EMR, which may led to further undertreatment of the patients [39]. It is expected that adverse events will decrease with the gain of experience [40,41,42], and more than 95% of the perforations are managed by endoscopy.

Other arguments that are pointed to be favorable to piecemeal EMR but are being challenged are procedural time, need for hospital admission, and costs. A large Swedish study showed excellent outcomes on performing colorectal ESD in an outpatient setting [43]. Furthermore, a large cohort found universal ESD for colorectal lesions above 20 mm to be more cost-effective than selective ESD or universal piecemeal EMR, due to the lower number of colonoscopies needed in the follow-up and the avoidance of a larger number of surgeries [44].

## 4. The Problematic of the Non-Curative ESDs (NC-ESD)

### 4.1. NC-ESD Definition

ESD is a technically challenging procedure, with a long learning curve [45,46]. Therefore, it is expected that, according to the experience of the endoscopist and the selection of the lesions, a significant number of procedures would not fulfill all the curative criteria.

The management of patients with a NC-ESD is not definitely established in any organ. The balance between referring the patient to major surgeries, with all the associated morbidity and mortality, must be always weighted with the risk of inclusion in surveillance programs only, missing the window of opportunity for complementary treatments (not only surgical but also systemic therapies) and therefore raising the risk for cancer progression and late metastatic disease development.

Current guidelines established the criteria for an ESD to be considered curative [12,13,23,37]. Generally, an en bloc, R0 resection of a benign lesion or of a well-to-moderately intramucosal or superficial submucosal adenocarcinoma (SM1 submucosal adenocarcinoma that is defined as the superficial third of the submucosal space and accepted to correspond to a submucosal depth, from the *muscularis mucosa* layer, of 200 μm in esophageal SCC, 500 μm in Barrett’s esophagus and gastric adenocarcinoma, and 1000 μm in colorectal cancer), without lymphovascular invasion, may be considered curative.

ESGE guidelines [12] suggested a new classification for ESD outcomes (Table 1). Curative resections refer to lesions removed in one single piece, with free horizontal and vertical margins, mucosal or submucosal without high-risk features, namely deep invasion (>sm1), lymphovascular invasion, poor differentiation or high-grade budding (with specificities depending on the organs, as seen below). These are “very-low-risk resection” (VLRR) and “low-risk resection” (LRR), lesions with very low (for example, intramucosal malignancy without high-risk factors) or low (such as submucosal adenocarcinoma without high-risk factors) probability of having lymph node metastasis (LNM). “Local-risk resection” (LocRRs) includes those with higher risk of having local recurrence (in the ESD site), such as those that are piecemeal-resected or with positive (benign) horizontal margins. “High-risk resection” (HRR) includes those with high-risk features of having LNM, warranting complementary treatment.

Additionally, some organ-specificities exist:

(a) For esophageal SCC, curability criteria may be stricter, due to the probable higher risk of LNM for the same staging comparing to other organs. Japanese and European guidelines consider an en bloc and R0 resection of a pT1a SCC without lymphovascular invasion curative, particularly if limited to the epithelium or *lamina propria* (m1 or m2); no evidence-based recommendation could be made for pT1a with *muscularis mucosa* invasion (m3), but generally no additional treatment is warranted, particularly in smaller lesions. There is no consensus regarding pT1bSM1 SCC: in well-differentiated lesions smaller than 2 cm without other risk criteria, the rate of LNM may be very small, so endoscopic follow-up (after proper staging) may be sufficient (LRR); nevertheless, Japanese guidelines suggest complementary treatment. Whenever other high-risk criteria are present (lymphovascular invasion, deep submucosal invasion, or positive vertical margin), adjuvant treatment is highly recommended.
(b) Regarding gastric neoplasia, a large study on surgical specimens from Japan found and validated a scoring system that aimed to help decision-making after an NC-ESD [47]. This score, the “eCura system”, included five risk factors for the development of LNM found in the ESD specimens, and weighed them according to the relative risk: three points for lymphatic permeation and one point each for lesion size above 30 mm, positive vertical margins, venous invasion and submucosal invasion equal or above 500 μm. Patients were categorized in three groups: low-risk group (0–1 point, 2.5% risk of LNM), intermediate group (2–4 points, 6.7% risk), and high-risk group (5–7 points, 22.7% risk). Accordingly, lesions removed in an en bloc fashion that follows one of these conditions are considered curative (eCuraA curative resection according to Japanese Guidelines and VLRR or LRR resections in European Guidelines): (1) Predominantly differentiated type, intramucosal (pT1a), non-ulcerated, with free horizontal and vertical margins and without lymphovascular invasion, regardless of the size; (2) Predominantly undifferentiated type, measuring 2 cm or less, intramucosal (pT1a), non-ulcerated, with free horizontal and vertical margins and without lymphovascular invasion; (3) Ulcerated, measuring 3 cm or less, predominantly differentiated type, intramucosal (pT1a), with free horizontal and vertical margins and without lymphovascular invasion. Lesions with superficial submucosal invasion (pT1bSM1), measuring 3 cm or less, predominantly differentiated type, with free horizontal and vertical margins and without lymphovascular invasion would also probably be curative (Japanese eCuraB, European LRR). If these criteria are not fulfilled, those will be non-curative resections and the likelihood of remnant lesion is high (eCuraC). If the only criterion among differentiated lesions that was not considered for being included in eCuraA or eCuraB was positive horizontal margin or piecemeal resection, these are eCuraC-1 lesions, and endoscopic follow-up could be considered due to a low risk of LNM, provided that the submucosal invasive part of the lesion was en bloc resected and with free margins, and the lesion was not ulcerated (European LocRR). All the others are eCuraC-2 lesions (European HRR) and complementary treatment is warranted (Table 2).
(c) Regarding colorectal lesions, ESD resections of benign lesions are curative if removed en bloc and R0 (VLRR); the others (piecemeal-resected or with positive horizontal margin, LocRR) should be managed by endoscopy. T1 (submucosal) carcinomas are considered radically removed if the following conditions are satisfied: free vertical margins, papillary or tubular adenocarcinoma, SM1 invasion, no lymphovascular invasion, and low-grade tumor budding (LRR). Endoscopic follow-up and treatment may be sufficient if removed in piecemeal or with positive horizontal margins (of a benign component—LocRR). Surgery is usually recommended if high-risk criteria are present (HRR), with the possible exception of deep submucosal invasion as the sole criterion, which may carry a low risk of LNM [48,49].

### 4.2. The Management of NC-ESD

Gathering valuable scientific evidence for the management of patients with NC-ESD is difficult, since the majority of the ESDs are curative, and among the non-curative procedures overall, the rate of LNM is low. This leads to a small number of outcomes (for example, LNM) among the total number of ESDs that are performed worldwide. There is a need for analyzing thousands of ESDs, to have a few hundreds of NC-ESDs; from them, only a percentage of cases will be selected (for example, malignant lesions). Then, only a few will present residual lesion or LNM that would allow statistical analysis and correlation with risk factors.

Therefore, multicenter, multinational collaboration is fundamental in order to obtain a sufficient amount of data that could contribute in the search of more adequate criteria. This is particularly important in the West due to the much lower number of ESDs compared to Asia; multinational collaborations have been created with this purpose [50,51,52,53,54,55,56].

#### 4.2.1. Esophagus

A Korean study [57] included 24 patients with esophageal SCC with submucosal invasion or with lymphovascular invasion and showed that the outcomes were similar between those followed-up without additional treatment and those with complementary surgery (5-year overall survival of 73% vs. 82%, *p* = 0.958 and 5-year recurrence of 25% vs. 43%, *p* = 0.388). A meta-analysis [58] on non-curative ESDs of esophageal SCC comprising Asian studies showed that complementary treatment with surgery may lead to less recurrences comparing to chemo/radiotherapy, with even higher recurrence rates in patients without any additional intervention. Additionally, deep submucosal invasion and lymphovascular invasion were independent factors for predicting recurrence in patients submitted to endoscopic resection and chemo/radiotherapy. Chemoradiation may, in fact, lead to a higher recurrence rate, but overall and disease-specific survival may be similar [59], and its efficacy has been prospectively demonstrated after endoscopic resection of pT1a Ly+ or pT1b R0 patients [60].

A recent study on Barrett’s neoplasia showed that vertical margin histological assessment may be challenging, and vertical margin status changed in nearly 25% of ESD cases after histological reassessment and did not necessarily imply residual lesion in the follow-up [61]. There are small studies showing good clinical outcomes of ESD in esophageal adenocarcinoma in the West, but with a small sample of NC-ESD [62]. Another recent study on pT1b adenocarcinomas showed that the majority had criteria for non-curability (86%), and 52% had no residual disease within the esophagectomy specimen [63]. Recent guidelines suggest that patients with high-risk pT1b adenocarcinomas (>SM1, poor differentiation or with lymphatic invasion) should be considered for further treatment (chemotherapy, radiotherapy or surgery) [64]. A recent Western multinational study from high-volume centers showed 8% (2 out of 26 operated patients) of LNM in the surgical specimen after an endoscopic resection of a vertical margin negative, high-risk T1 esophageal adenocarcinoma [65]. In contrast, another study including patients with positive vertical margin showed 50% of residual lesion [61]. Despite these, these numbers are still not sufficient to draw conclusions about the risk factors concerning lesion characteristics and pathological findings on ESD specimens, such as deep submucosal invasion, poorly differentiated cancers, or lymphovascular invasion, for the presence of LNM after an HRR.

#### 4.2.2. Stomach

There are some pre-ESD features (lesion location, morphology, and pathology) that predict the risk of a non-curative ESD requiring gastrectomy [66]. After ESD, the challenge is, again, to find accurate predictors, based on the pathological examination of the ESD specimen, of those patients who would benefit from complementary treatment. There are some data showing that, overall, patients who underwent surgery after a NC-ESD have a better overall survival and, sometimes, cancer-specific survival, compared to those are only followed-up without additional rescue treatment [67]. However, significant selection bias may be present, due to the data’s retrospective nature and the heterogeneity of non-curative criteria.

The eCura system [47] establishes a stratification for LNM. There are some studies that have further validated [68] and even modified this system [69], and it has been suggested that cancer recurrence and cancer-specific mortality are higher in patients with no additional treatment compared to those with radical surgery, in patients classified in the eCura high-risk category [70], whereas similar outcomes were found for patients in the intermediate- and low-risk groups [71]. On the other hand, in local-risk resections (positive lateral margin), additional endoscopic treatment within 3 months could improve disease-free survival [72].

A recent study performed an external validation of the eCura score in the West, showing its accuracy in this population. Additionally, a slight modification of this score was proposed (the W-eCura score), changing the submucosal invasion cut-off (SM1) from 0.5 mm to 1 mm, leading to an even higher accuracy (AUROC 0.916, 95% CI 0.870–0.961), maintaining a high sensitivity (92%) [52]. In this large multinational study, it was noteworthy that 68% of the patients who underwent surgery due to a NC-ESD did not present residual lesion in the gastric wall or in the lymph nodes. The indication for surgery due to oncological ESD failure remains far from definite [73], and lymphatic invasion must be the strongest risk factor for LNM [74], together with deep submucosal invasion, size, and undifferentiated histology [12]. For local recurrence at the ESD site, other criteria such as positive horizontal or margins are also implied as risk factors [75,76].

#### 4.2.3. Colon and Rectum

Regarding colorectal NC-ESD in the West, data are scarce. A recent study showed that overall mortality due to non-colorectal cancer causes may be higher among patients submitted to follow-up compared to patients that underwent surgery, but with similar tumor recurrence and disease-specific survival rates [51]. However, no risk factors for LNM or recurrence were addressed, and the follow-up time may be too short for tumor recurrence and survival analyses. Regarding LocRRs of colorectal ESD, a Western multinational study (R-ESD study) [50] analyzed 354 lesions, being the largest cohort on ESDs with positive horizontal margins after a complete ESD resection. It was found that the residual rate after a median follow-up of 24 months was similar, and very low (2.1%), for patients with positive horizontal margins, comparing to 0.5% in the control group (negative horizontal margins), considering benign lesions only (*n* = 308) (*p* = 0.251). This study supports the idea that most of the positive horizontal margins that are reported after a colorectal ESD are false positives. Similarly, another large multinational study on LocRR found a higher rate of recurrence after a piecemeal resection, but not after an en bloc resection with positive horizontal margins [53].

A multinational study on malignant (pT1) lesions with a non-curative resection (*n* = 135 among 2255 colorectal ESDs) [54] found an 18% rate of residual lesion overall: an LNM rate of 14% (among operated patients) and 13% of residual lesion in the colorectal wall. Two predictive scores were proposed: NC-Lymph score for predicting LNM (lymphatic permeation scoring 2 points and poor differentiation with 1 point) and NC-Wall score for predicting residual disease in the ESD site (positive vertical margin and poor differentiation scoring 2 points each, and piecemeal resection scoring 1 point). An NC-Lymph score higher than 0 warrants surgery due to LNM risk of 27% or higher, while an NC-Wall score of 0 has a very low risk of residual lesion in the wall. In this study, the vast majority of patients submitted to surgery (79%) had no residual lesion in the wall or LNM.

The best cutoff for defining deep submucosal invasion for LNM has been debated, with sm3 having a higher risk of LNM comparing to sm1 and sm2 in a metanalysis [77], while other studies failed to show deep submucosal invasion as an independent factor for LNM [78], or only significant with invasion deeper than 2 mm [79]. The controversial significance of this parameter as a substantial risk factor for metastasis has led to the development of novel techniques derived from ESD, as Endoscopic Intermuscular Dissection in the rectum [80], where lesions invading deep layers of submucosa or even the superficial *muscularis propria* may be resected by endoscopy, with 45% of curative resections [81]; further follow-up is needed to address oncological outcomes of these patients in the mid and long term.

Still, in the case of negative deep margin, disease-specific survival or recurrence-free survival may be lower in the patients followed-up without additional surgery in the presence of high-risk criteria such as lymphatic invasion or high-risk budding, while survival rates may be similar between NC-ESDs with surgery compared to curative endoscopic resections [82].

## 5. New Technologies and Future Perspectives

It is clear that the management of patients after an NC-ESD is under continuous debate. Therefore, in the absence of definite orientations, the decision on whether performing additional oncological treatment should draw a balance between the risk of disease progression and the morbidity and mortality of further interventions, always taking in consideration patient age and medical status; in fact, algorithms that include not only pathological criteria but also patient factors have already been created [67]. Furthermore, the continuous technology research and innovations may be helpful in this subject.

Artificial intelligence is being increasingly applied in gastrointestinal endoscopy [83]. In the field of ESD, it has been tested, for example, to assist in delineating the lateral margin of gastric lesions [84] and to predict the post-procedural bleeding risk [85] or the risk of LNM in early gastric cancer [86]. In the case of gastric cancer, there are already machine learning models that surpass the well-established e-Cura score for predicting LNM in early gastric cancer not meeting endoscopic curability criteria [87]. In the future, artificial intelligence systems may help in the pre-procedural phase, aiding in the selection of the lesions amenable to ESD and differentiating gastric intramucosal from submucosal invasive cancers [88], as well as invasion depth throughout the gastrointestinal tract [89], in the ESD phase, helping increasing the rate of a curative resection and lowering adverse events, for example, by aiding in the coagulation of vessels on the ESD bed [90]; they may also help in the post-procedural phase, in which, together with endoscopic evaluation and pathological assessment, they may perhaps increase the accuracy in the management of patients with a non-curative ESD and consequent referral to surgery.

What seems unquestionable is that referring patients with a non-curative ESD to surgery based on single criteria leads to a very high rate of futile surgeries. Furthermore, the diverse histological criteria that define a non-curative ESD do not surely have the same weight regarding residual risk and oncological progression. Most of the criteria that are considered to be high risk for LNM came from studies that are not recent, have a limited number of patients, and are based on surgical specimens; moreover, in some locations like in Barrett’s esophagus, studies in this context are mainly in EMR specimens instead of ESD resections. The main challenge in having more definite conclusions is related to the difficulty of having a large amount of data, due to the overall low incidence of residual lesions or LNM among NC-ESDs, which precludes the establishment of definite risk factors or predictive scores.

Better definitions and cut-offs of individual criteria need to be continuously pursued, as the decision must result from a weighted combination of the different criteria in clinical algorithms, in order to offer the patients the most accurate prediction of residual neoplasia. Long-term, prospective, and collaborative multinational studies are needed in order to surpass the difficulty of having enough data to manage patients through an evidence-based approach.

## Figures and Tables

**Table 1:** Curativeness definition according to ESGE guidelines.

Resection	ESGE Classification	Features	Interpretation
Curative	VLRR	Lesions en bloc removed, with free horizontal margins, mucosal (VLRR) or submucosal (LRR), without high-risk features.	Very low risk of LNM
	LRR		Low risk of LNM
Non-curative	LocRR	Piecemeal-resected benign lesions, positive horizontal margins of benign component, without high-risk features.	High risk of local recurrence
	HRR	Presence of high-risk features: Lymphovascular invasion, poor differentiation, deep submucosal invasion, positive vertical margins	High risk of LNM

VLRR: Very-low-risk resection. LRR: low-risk resection. LocRR: local risk resection. HRR: high-risk resection. LNM: lymph node metastasis.

**Table 2:** e-Cura system for curability of gastric ESDs, and its relationship with ESGE nomenclature.

Resection	ESGE Classification	e-Cura	Features
Curative	VLRR/LRR	A	predominantly differentiated type, intramucosal, non-ulcerated, with free horizontal and vertical margins and without lymphovascular invasion, regardless of the size; predominantly undifferentiated type, measuring 2 cm or less, intramucosal, non-ulcerated, with free horizontal and vertical margins and without lymphovascular invasion; ulcerated, measuring 3 cm or less, predominantly differentiated type, intramucosal, with free horizontal and vertical margins and without lymphovascular invasion
	LRR	B	Lesions with superficial submucosal invasion (pT1bSM1), measuring 3 cm or less, predominantly differentiated type, with free horizontal and vertical margins and without lymphovascular invasion
Non-curative	LocRR	C-1	If the only criteria among differentiated lesions that was not respected for being included in eCuraA or eCuraB were positive horizontal margin or piecemeal resection, provided that the submucosal invasive part of the lesion was en bloc resected and with free margins, and the lesion was not ulcerated
	HRR	C-2	Lesions not fulfilling other groups’ criteria

## Data Availability

Not applicable.

## Abbreviations

EMR	Endoscopic Mucosal Resection
ESD	Endoscopic submucosal dissection
LNM	Lymph node metastasis
LST	Lateral Spreading Tumor
NC-ESD	Non-curative ESD
POEM	PerOral Endoscopic Myotomy
SCC	Squamous cell carcinoma
